# Hepatoprotective role of peroxisome proliferator-activated receptor-α in non-cancerous hepatic tissues following transcatheter arterial embolization

**DOI:** 10.1515/biol-2022-0068

**Published:** 2022-08-11

**Authors:** Peiyu Yang, Zhengliang Li, Wei Du, Chunhua Wu, Wencui Xiong

**Affiliations:** School of Clinical Medicine, Dali University, Dali City, Yunnan Province 671000, China; Department of Radiology, The First Affiliated Hospital of Dali University, No. 32, Jiashibo Street, Dali City, Yunnan Province 671000, People’s Republic of China

**Keywords:** rabbit VX2 hepatic carcinoma, transcatheter arterial embolization, peroxisome proliferator-activated receptor-α, oxidative stress, inflammation, apoptosis

## Abstract

Transcatheter arterial embolization (TAE) is a widely used technique in treating hepatic carcinoma but may cause liver injury in some cases. This study investigated the hepatoprotective effect of the preprocessed peroxisome proliferator-activated receptor-α (PPAR-α) agonist-WY-14643 following TAE. A total of 60 rabbit liver cancer models were developed and divided into a combined treatment (WY-14643 and TAE), TAE, and control groups. After TAE, we examined the histopathological picture and liver functions. Further, the expression of antioxidant enzymes, tumor necrosis factor-α (TNF-α), nuclear factor of κ-light chain of enhancer-activated B cells (NF-κB), PPAR-α, and B-cell lymphoma-2 (Bcl-2) was analyzed. Liver function tests, pathology score, and apoptosis index significantly worsened in the TAE group but were normalized in the combined treatment group. In addition, ELISA results showed that antioxidant enzyme activity significantly increased, while the malondialdehyde content and level of inflammatory cytokines were significantly reduced in the combined treatment group. Furthermore, compared to the TAE group, the expressions of PPAR-α, antioxidant enzymes superoxide dismutase1 (SOD1) and SOD2, and Bcl-2 were significantly elevated, while NF-κB was significantly reduced in the combined treatment group. On the other hand, the expression of NF-κB in tumor tissues was significantly reduced by pretreatment with WY-14643. Therefore, PPAR-α can ameliorate liver injury by exerting its anti-oxidative, anti-inflammatory, and anti-apoptotic functions.

## 1 Introduction

The annual death toll caused by primary liver cancer (PLC) is around 782,000 deaths, making it the second leading cause of cancer-related mortality worldwide [1]. In China, PLC is the fourth most common malignancy and the third leading cause of tumor-related deaths [2,3,4]. Hepatocellular carcinoma (HCC) is the most common type of PLC (90% of all cases), for which treatment strategies include liver transplantation, hepatic resection, and image-guided ablation depending on the age and overall health condition of the patient [5]. Current guidelines recommend transcatheter arterial embolization (TAE) for unresectable advanced liver cancer cases [6,7,8]. HCCs are primarily perfused by the hepatic artery, which supplies ∼20% blood to liver, providing a rationale to consider embolization of this artery as a strategy for selective ischemia and tumor size reduction [9]. However, approximately 30–80% of patients develop hepatic ischemia after TAE [10], which induces oxidative stress, inflammation, apoptosis, and necrosis [11]. Given that 80% of PLC patients are presented with liver cirrhosis [12], additional liver injury can compromise the patient’s quality of life. Therefore, developing strategies that can reduce adjacent liver tissue damage after TAE can positively improve a patient’s prognosis.

Peroxisome proliferator-activated receptor-alpha (PPAR-α), a subtype of the ligand-activated PPARs family, is pertinent in the transcriptional regulation of genes involved in lipid and carbohydrate metabolism and various cellular functions, including proliferation, death, and differentiation. PPAR-α also plays a crucial role in inflammation, angiogenesis, and immune response and is predominantly expressed in the liver [13]. Echeverría et al. previously demonstrated that the PPAR-α pathway can upregulate nuclear factor erythroid 2-related factor-2 (Nrf2) to increase the cellular antioxidant potential and downregulate nuclear factor of κ-light chain of enhancer-activated B cells (NF-κB) in nonalcoholic fatty liver disease (NAFLD) [14]. Therefore, PPAR-α can inhibit oxidative stress, inflammation, and apoptosis, which is important in the improvement of alcoholic fatty liver disease (ALD) as well as hepatitis B viral infection [15,16]. However, it remains to be verified whether PPAR-α can exert anti-oxidative, anti-inflammatory, and anti-apoptotic effects in non-cancerous liver tissues following TAE. Pirinixic acid (PA, WY-14643) is a PPAR-α agonist that was originally developed to treat hyperlipidemia with hepatoprotective effects against ischemic injury [17]. In this study, we examined the protective role of the PPAR-α agonist, WY-14643, in decreasing the oxidative stress, inflammation, and apoptosis in the adjacent liver tissue following TAE in a rabbit model of liver cancer.

## 2 Materials and methods

### 2.1 Animals

A total of two VX2 tumor rabbits were provided by the Cancer Hospital Affiliated to Nanjing Medical University. Seventy healthy New Zealand white rabbits were purchased from Kunming Chushang Technology Co., Ltd, license number: SCXK (Yunnan) K20180001. The body weight of rabbits, including both males and females, ranged from 2.0 to 3.50 kg (average of 2.85 ± 0.25 kg), and all animals were fed a diet of rabbit pellets.


**Ethical approval:** The research related to animal use has been has been complied with all the relevant national regulations and institutional policies for the care and use of animals. The study protocol was approved by the Biomedical Ethics Committee of Dali University, China.

### 2.2 Establishment of rabbit VX2 liver cancer model

**Figure 1:**
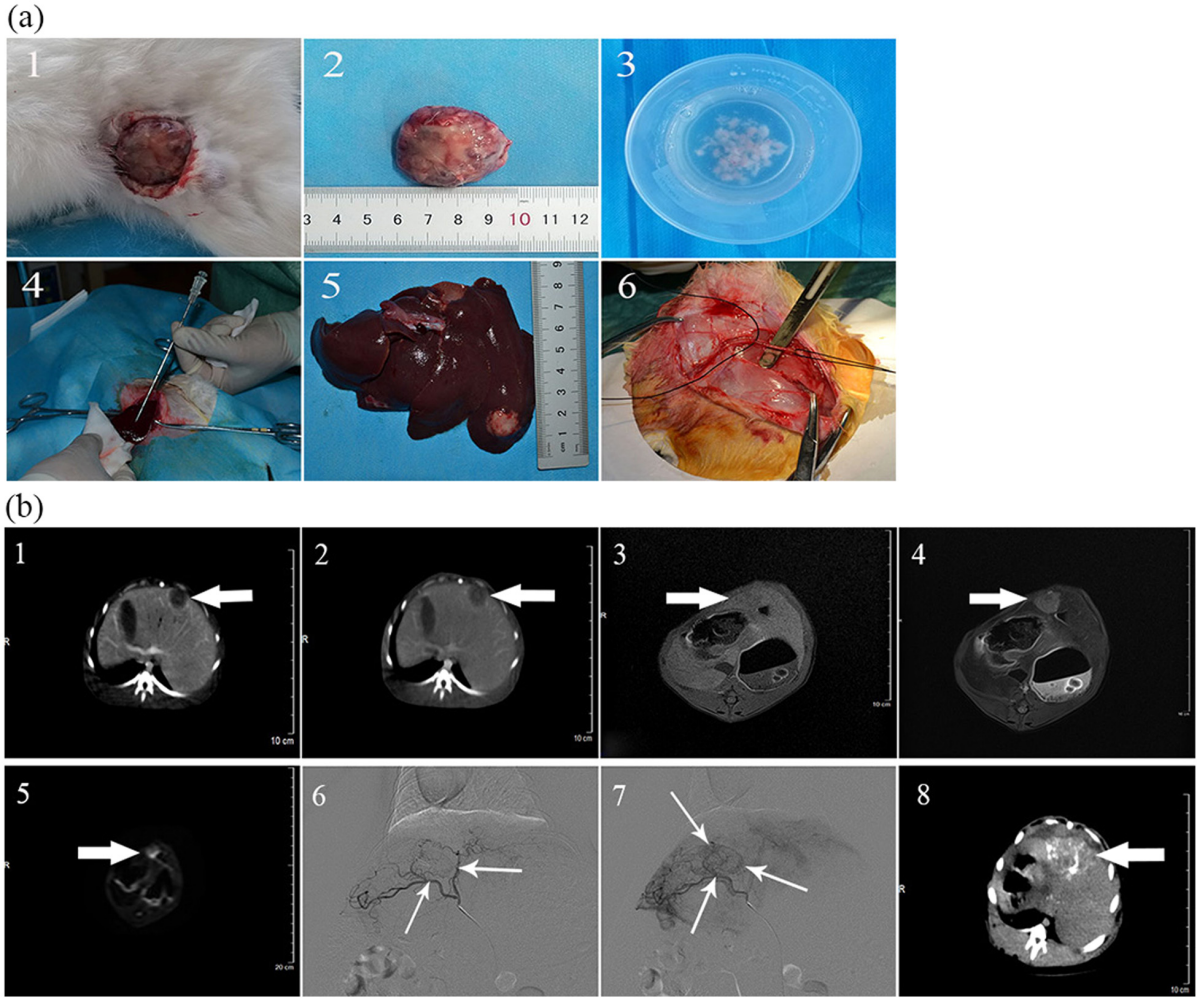
(a) Establishment of liver cancer model and separation of femoral artery during TAE: (1) pictogram demonstrating the isolation of tumor tissue from the hind legs of tumor-bearing rabbits; (2) pictogram demonstrating isolated tumor in rabbit’s hind leg; (3) pictogram showing the fragmentation of the tumor tissue; (4) pictogram demonstrating planting small tumor fragments into the left lobe of the rabbit liver; (5) pictogram demonstrating successful induction of liver cancer; and (6) separation of femoral artery during TAE. (b) CT and MRI of rabbit VX2 liver cancer model (*n* = 20): (1) solid part of the mass obviously strengthened in arterial phase; (2) significantly reduced degree of enhancement of the mass in the portal phase; (3) mass with a low signal in the T1WI fat suppression sequence; (4) mass with a slightly high signal in the T2WI fat suppression sequence; (5) mass with an obviously high signal in DWI. TAE of rabbit VX2 liver cancer model (*n* = 20): (6) early arterial of TAE; (7) blood vessels around the tumor showing a bulge sign in late arterial of TAE; and (8) lipiodol deposition in tumor after TAE.

We established the rabbit liver cancer model by injecting tumor cells into healthy rabbits as detailed previously [18]. Briefly, we anesthetized one tumor-bearing rabbit by intravenous injection of 3% pentobarbital sodium (30 mg/kg), extracted the tumor, then removed the necrotic and fibrous tissues (Figure 1a(2)). The tumor was cut into 1 mm^3^ pieces (Figure 1a(3)) in 0.9% sodium chloride and 20,000 U gentamicin solution and dispersed with a 1 mL syringe. Next the tumor particle mixture (15–20 tumor particles/mL) was injected into the hind leg of a healthy rabbit to create more tumor-bearing rabbits. Alternatively, following anesthesia and abdominal incision, the liver was exposed, and 1 mL of tumor particles was injected into the left live lobe at a 30° angle to create the liver cancer model (Figure 1a(4)). The puncture channel was filled with a gelatin sponge, and gauze was used to compress the puncture point for 3–5 min to achieve hemostasis. Next the abdominal cavity was irrigated with gentamicin (4,00,000 U), then the incision was sutured. Each rabbit was administered intramuscular injections of gentamicin (4,00,000 U) for 3 consecutive days to prevent infection. At 7 and 14 days after surgery, tumor implantation and growth were monitored with computed tomography (CT) and magnetic resonance imaging (MRI) using a 16-row spiral CT scanner (Philips Healthcare) and VANTAGE TITAN 3.0T MRI (Toshiba Inc.), respectively.

### 2.3 Experimental ground and treatment modules

A total of 60 rabbit VX2 liver cancer models were successfully established. The rabbits were randomly divided into 3 experimental groups, including a control group (*n* = 20), TAE group (*n* = 20), and combined treatment group (WY-14643 + TAE; *n* = 20). Rabbits in the combined treatment group underwent TAE interventional surgery and received 3 mg/kg/day WY-14643 (Sigma, USA) via the marginal ear vein for 3 consecutive days before operation. Rabbits of the TAE group underwent TAE interventional therapy and were given 8 mL/kg/day of 10% dimethyl sulfoxide (DMSO; Tianjin Chemical Reagent Co., Ltd, China) through the marginal ear vein for 3 consecutive days. Similarly, rabbits in the control group were administered with 10% DMSO for 3 days and received no other treatments. Two radiologists performed TAE using digital subtraction angiography (Integris Allura 12, Philips, Netherlands), as previously described [19]. Briefly, an 18G puncture needle was used to penetrate the left femoral artery (Figure 1a(6)), then a 4F catheter sheath and catheter were introduced. The abdominal trunk was selected through the guide wire, and a 3F microcatheter system was utilized to select the common hepatic artery. Next we injected a contrast agent to confirm the tumor supply artery (Figure 1b(6–7)) and subsequently inserted ethiodized oil (0.15 mL/kg) through the microcatheter. The dose of ethiodized oil was adjusted to ensure that the tumor blood flow slowly stopped and that the tumor supply arteries were embolized with gelatin sponge particles. Three days after TAE, the rabbits underwent CT scanning to enable the comparison of the post-operative liver images with the baseline images.

### 2.4 Determination of liver function

Rabbits were euthanized 3 days after TAE, and the blood samples (4 mL per rabbit) were obtained from the ear vein to assess the liver function. Serum alanine transaminase (ALT) and serum aspartate transaminase (AST) were determined using ALT/GPT and AST/GOT test kits (Shanghai Boyao Biotechnology Co., Ltd, China), according to the manufacturer’s protocol.

### 2.5 Analysis of liver histopathology

At the end of the experiment (day 3 post-TAE), rabbits were sacrificed by injecting 10 mL of air through the ear vein. Liver tissues 2 cm away from the tumor edge were obtained for conventional Hematoxylin and Eosin (HE) staining to measure the liver histology score, as previously described [20]. Briefly, liver tissues were fixed, dehydrated, embedded in paraffin, and sectioned. Then, the sections were deparaffinized, rehydrated, and stained with HE (Sigma, USA).

### 2.6 TUNEL staining

TUNEL staining was performed with a TUNEL kit (Yisheng Biotechnology Co., Ltd, Shanghai, China) following the manufacturer’s protocol. The number of apoptotic hepatocytes with brown or tan nuclei was counted on an optical microscope and used to calculate the apoptosis index (AI).

### 2.7 Detection of oxidative stress and inflammatory cytokines

Rabbit superoxide dismutase (SOD), catalase (CAT), and glutathione peroxidase (GSH-Px) kits, and malondialdehyde (MDA) test kits were purchased from Nanjing Jiancheng Bioengineering Institute Co., Ltd. Rabbit tumor necrosis factor-α (TNF-α) and nuclear factor of κ-light chain of enhancer-activated B cells (NF-κB) p65 ELISA kits were purchased from Shanghai Lanji Biotechnology Co., Ltd. Liver tissue homogenates were prepared in ice-cold 0.9% sodium chloride solution, then oxidative stress-related indices (MDA, SOD, CAT, and GSH-Px) and inflammatory cytokines (TNF-α and NF-κB p65) were measured in accordance with the manufacturer’s protocol.

### 2.8 Relative gene expression of PPAR-α, SOD1, SOD2, NF-κB p65, and B-cell lymphoma-2 (Bcl-2)

RNA was extracted with TRIzol (Invitrogen, USA) and reverse transcribed with the RevertAid First Strand cDNA Synthesis kit (Thermo Scientific, USA) following the manufacturer’s protocol. Real-time PCR was performed using FastStart Universal SYBR Master (ROCHE, Switzerland). The relative expression of each target gene was determined by the comparative threshold method (2^−ΔΔCt^) normalized to the house keeping gene, GAPDH. Primer sequences are detailed in Table 1.

**Table 1:** Primer sequences

Primer	Sequences
PPAR-α	5′–CAG ATG GCT CCG TGA TCA CAG–3′ (forward)
	5′–ACC AGC TTT AGC CGA ATC GTT C–3′ (reverse)
SOD1	5′–CAC CAT CCA CTT CGA GCA GA–3′ (forward)
	5′–GTC ACA TTA CCC AGG TCG CC–3′ (reverse)
SOD2	5′–TGG ACA AAC CTG AGC CCT AAC–3′ (forward)
	5′–TCA CTT TCT GCA GGC CAA GT–3′ (reverse)
Bcl-2	5′–GGG TAT CCT TTC GAC GGC AA–3′ (forward)
	5′–GGC GTT TCC AAA GTA CGT GG–3′ (reverse)
NF-κB p65	5′–ACTTCCTGGCGCATCTAGTG–3′ (forward)
	5′–CATGTCCTTGGGTCCAGCAT–3′ (reverse)
GAPDH	5′–GCT GCT TTT AAC TCT GGC AAA GT–3′ (forward)
	5′–TGA TGG CCT TCC CGT TGA TG–3′ (reverse)

### 2.9 Relative protein expression of PPAR-α, SOD1, SOD2, and Bcl-2

Total proteins were obtained from liver tissues adjacent to the cancer region by a protein extraction kit (Beyotime, China). Protein samples were then separated using 12% sodium dodecyl sulfate-polyacrylamide gel electrophoresis (SDS-PAGE) and transferred onto nitrocellulose membranes. Membranes were blocked with 5% skim milk in TBST and incubated overnight at 4°C with the following primary antibodies: rabbit anti-PPAR-α (1:1,000, #ab24509, Shanghai, China), rabbit anti-SOD1 (1:1,000, Abcam Biotechnology, UK), rabbit anti-SOD2 (1:1,000, Abcam Biotechnology, UK), rabbit anti-Bcl-2 (1:1,000, #12789-1-AP, Wuhan, China), and rabbit anti-β-actin (1:1,000, Abcam Biotechnology, UK). On the following day, membranes were incubated with horseradish peroxidase-conjugated goat-anti-rabbit secondary antibodies (Boster Biological Technology, Wuhan) at room temperature for 2 h. An enhanced chemiluminescence (ECL) reagent (Thermo Fisher) was used to visualize the immunoreactive proteins. Signal densitometry was quantified by software analysis (Quantity One, Bio-Rad, USA).

### 2.10 Statistical analysis

All statistical analyses were performed on GraphPad Prism 7. Data are expressed as mean value ± standard deviation (*x̅* ± *s*). One-way ANOVA was used to compare the means of the three groups, and Tukey’s test was utilized to perform multiple comparisons between groups. A *P* value less than 0.05 was considered statistically significant.

## 3 Results

### 3.1 Establishment of liver cancer model and TAE

A total of 60 liver cancer models without metastasis were established (Figure 1a(5) and 1b(1–5)). Following TAE, the limited deposition of lipiodol in the blood supply area of tumors revealed successful TAE (Figure 1b(8)).

### 3.2 Assessment of liver function

**Figure 2:**
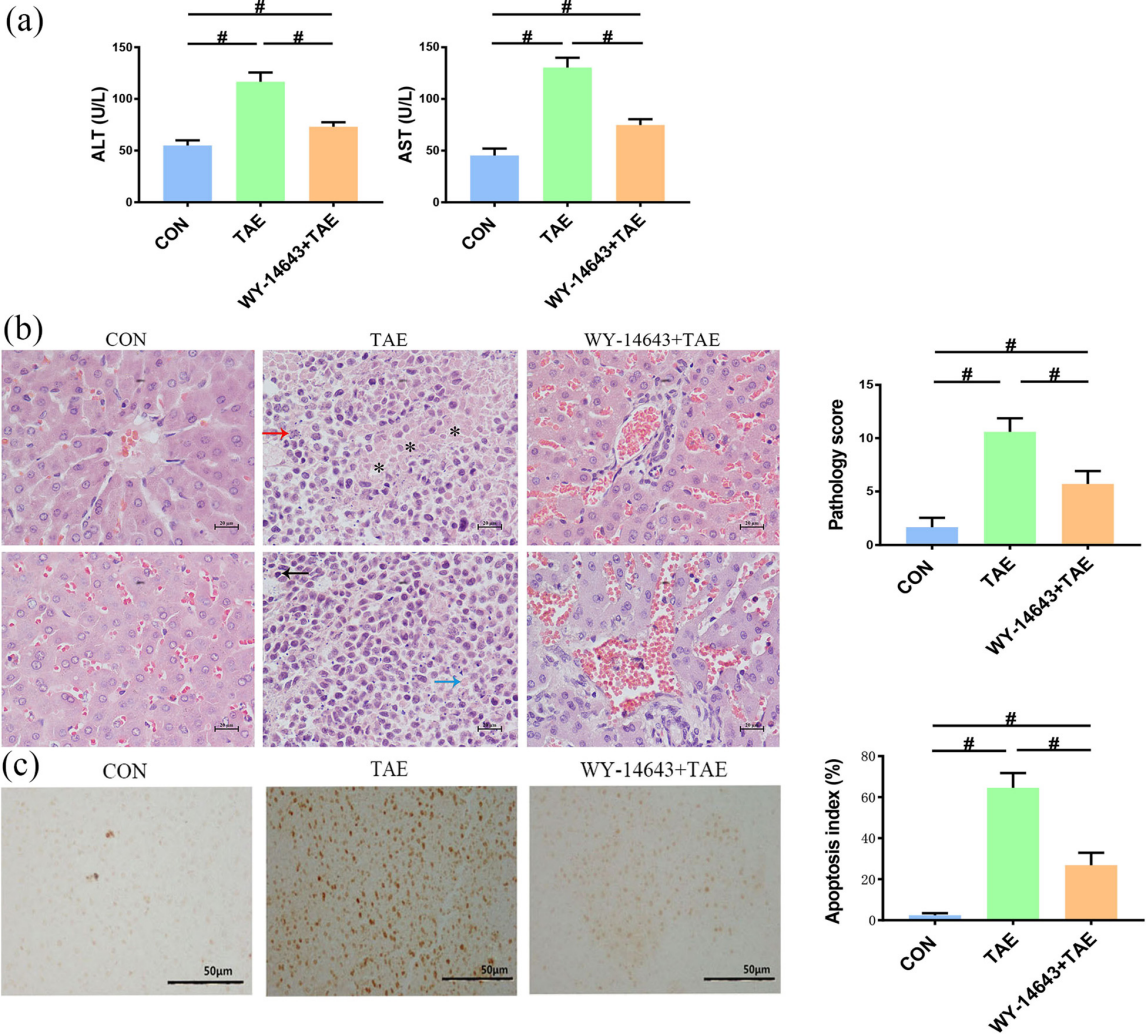
(a) Analysis of liver function. (b) Representative HE (HE) staining and corresponding pathology score among the different experimental groups (*n* = 20, HE, ×400, black arrow--nuclear pyknosis, red arrow--nuclear fragmentation, blue arrow--nuclear dissolution, and asterisk--large areas of red stained coagulative necrosis). (c) Hepatocyte apoptosis. Control group shows a few apoptotic cells distributed in scattered spots. TAE group exhibits a large number of apoptotic cells with deep staining and concentrated distribution. Combined treatment group demonstrates a fewer number of apoptotic cells with lighter staining and uneven distribution. ^#^Significant (*P* < 0.05).

Compared to the control group, the ALT and AST levels significantly increased in the TAE group (*P* < 0.001). Similarly, ALT and AST levels were significantly increased in the combined treatment group (*P* < 0.001) compared to the control group but significantly decreased compared to the TAE group (*P* < 0.001, Figure 2a).

### 3.3 Histopathological changes

HE staining was used to compare the histopathological changes among the experimental groups. Results revealed normal hepatic cytoarchitecture with no obvious abnormalities in the control group. In the TAE group, hepatocytes were necrotic with signs of nuclear shrinkage, nuclear fragmentation, or disappearance, while red cytoplasmic staining and hepatocellular edema were accompanied by light cytoplasmic staining and structural disorder. In the combined treatment group, liver tissue was rather normal with slight neutrophil infiltration and mild congestion. The mean value of the liver histology score significantly increased in the TAE group, and this increase was normalized in the combined treatment group (*P* < 0.05, Figure 2b).

### 3.4 Hepatocyte apoptosis

In order to evaluate the damage to non-cancerous tissues, we measured the apoptotic index of liver cells. The TAE group had a significantly higher number of apoptotic cells than the other two groups (Figure 2c). Compared with the control group, the AI of the TAE group and combined treatment group significantly increased (*P* < 0.001). Compared to the TAE group, AI of the combined treatment group was significantly reduced (*P* < 0.001, Figure 2c). These results indicate that non-cancerous tissues can be damaged by TAE treatment.

### 3.5 Assessment of oxidative stress in liver tissues

**Figure 3:**
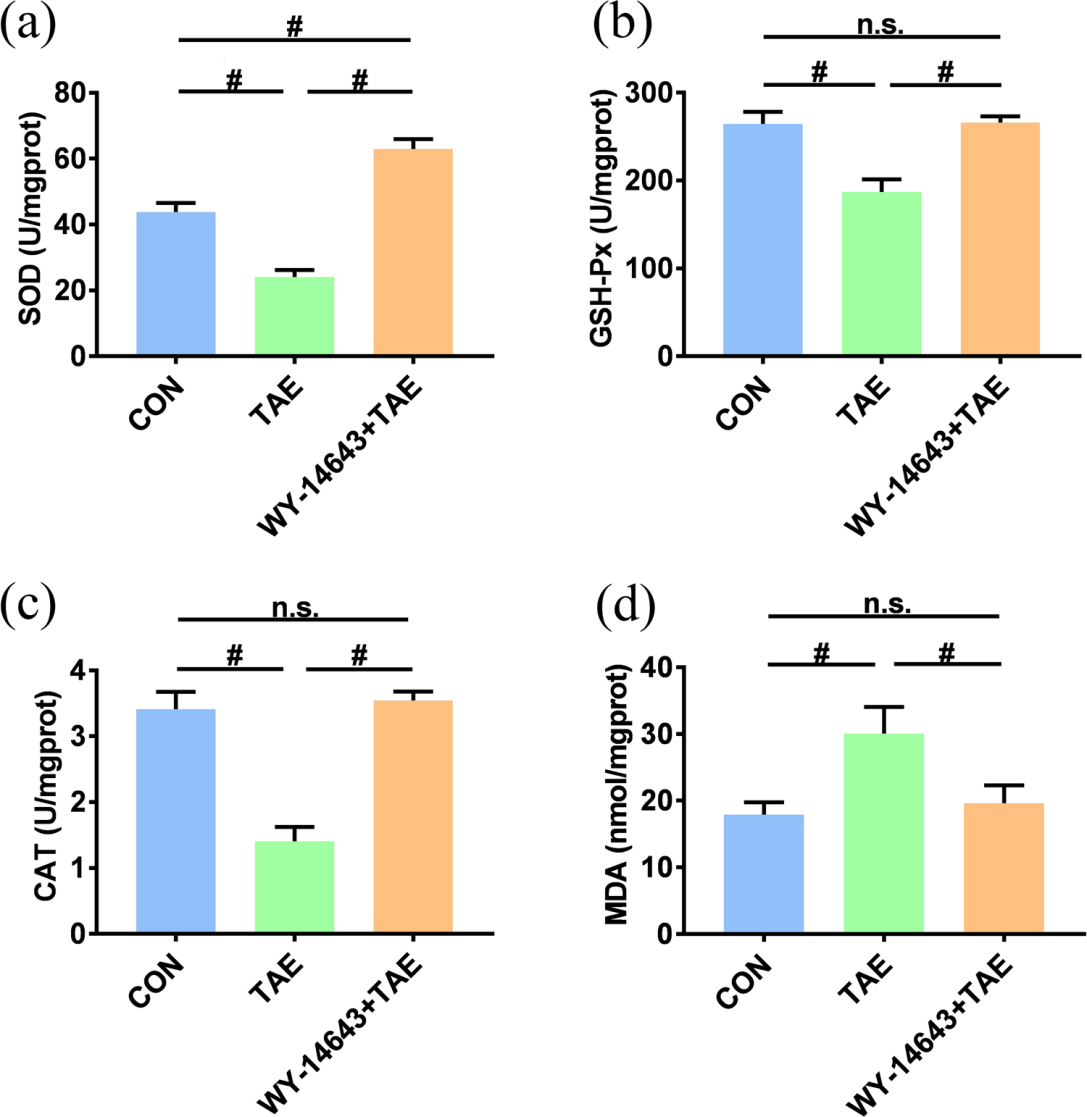
Bar charts representing the superoxide dismutase, glutathione peroxidase, catalase, and malondialdehyde levels among the different experimental groups (*n* = 20). ^#^Significant (*P* < 0.05), Not significant (n.s., *P* > 0.05).

Compared to the control group, the MDA level of the TAE group was significantly increased, while the activities of SOD, GSH-Px, and CAT were significantly reduced (*P* < 0.05). Compared to the TAE group, MDA level was significantly reduced in the combined treatment group, while the SOD, GSH-Px, and CAT activity significantly increased (*P* < 0.05). On the other hand, compared to the control group, the SOD activity was significantly increased in the combined treatment group (*P* < 0.05), while GSH-Px and CAT activity and MDA level did not change significantly (*P* > 0.05, Figure 3). These results suggest that oxidative stress occurred in the non-cancerous tissues after TAE.

### 3.6 Expression of inflammatory mediators in liver tissues

**Figure 4:**
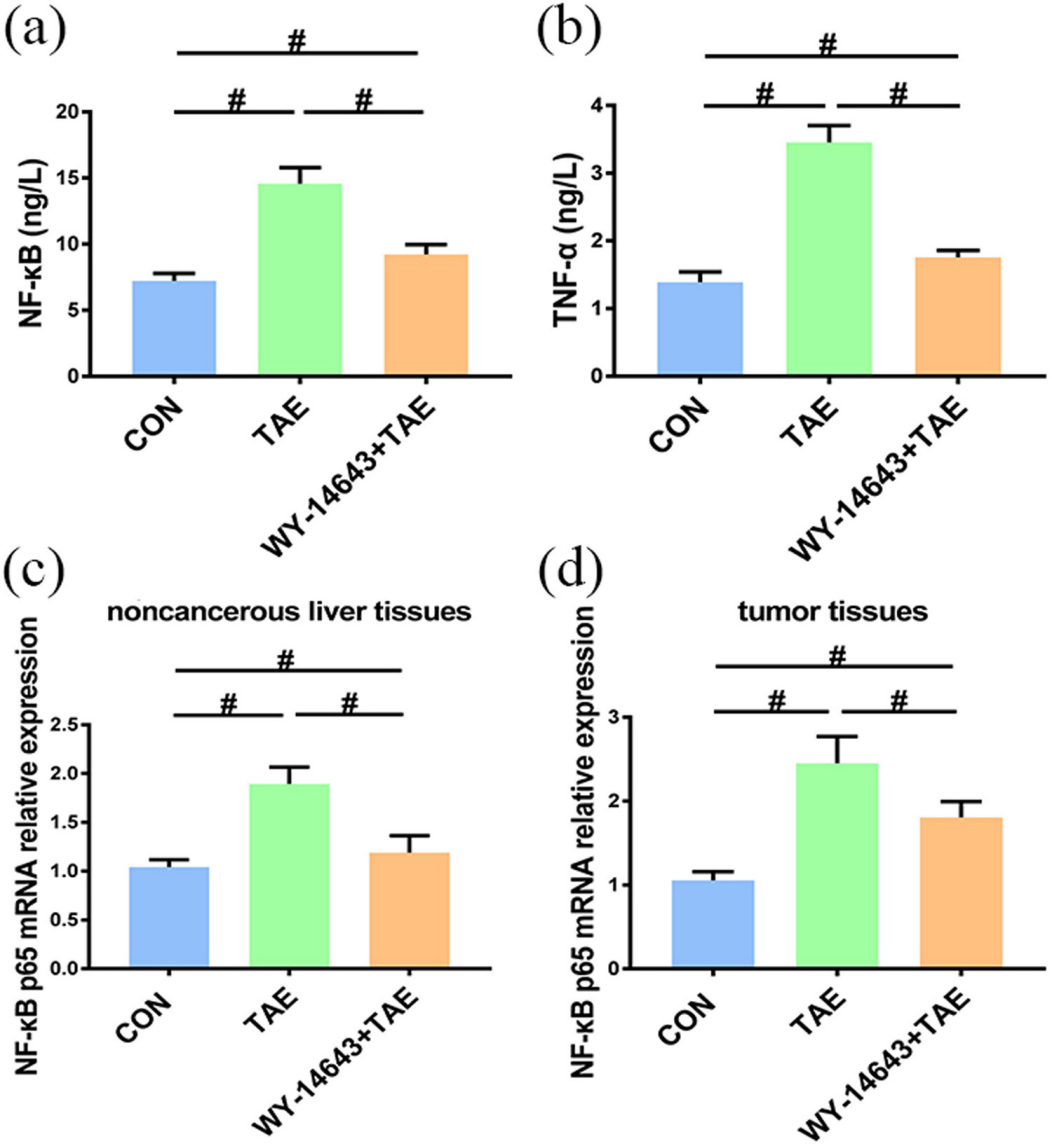
Bar charts show the nuclear factor of κ-light chain of enhancer-activated B cells and tumor necrosis factor-α levels among the different experimental groups (*n* = 20). (a and b) TNF-α and NF-κB levels in non-cancerous liver tissues detected by ELISA. (c and d) Gene expression of NF-κB in non-cancerous liver tissues and tumor tissues were detected by RT-qPCR. ^#^Significant (*P* < 0.05).

We measured NF-κB p65 and TNF-α levels to evaluate the inflammatory response of the liver tissues adjacent to the cancer tissues. ELISA results showed that the level of NF-κB p65 and TNF-α were significantly increased following TAE. This was significantly improved by WY-14643 pretreatment (*P* < 0.05, Figure 4a and b). Further, the relative gene expression of NF-κB p65 was significantly increased in the TAE group and significantly decreased in the WY-14643 pretreatment group (*P* < 0.05, Figure 4c). These results suggest that PPAR-α agonist WY-14643 can possibly alleviate inflammation of non-cancerous liver tissues following TAE.

### 3.7 Gene expression of NF-κB in tumor tissues

Similarly, the relative expression of NF-κB p65 gene was significantly increased in tumor tissues following TAE and significantly decreased in the WY-14643 pretreatment group (*P* < 0.05, Figure 4d). The above results indicated that WY-14643 can also modulate NF-κB expression in cancerous tissues following TAE.

### 3.8 PPAR-α, SOD1, SOD2, and Bcl-2 relative gene expression

**Figure 5:**
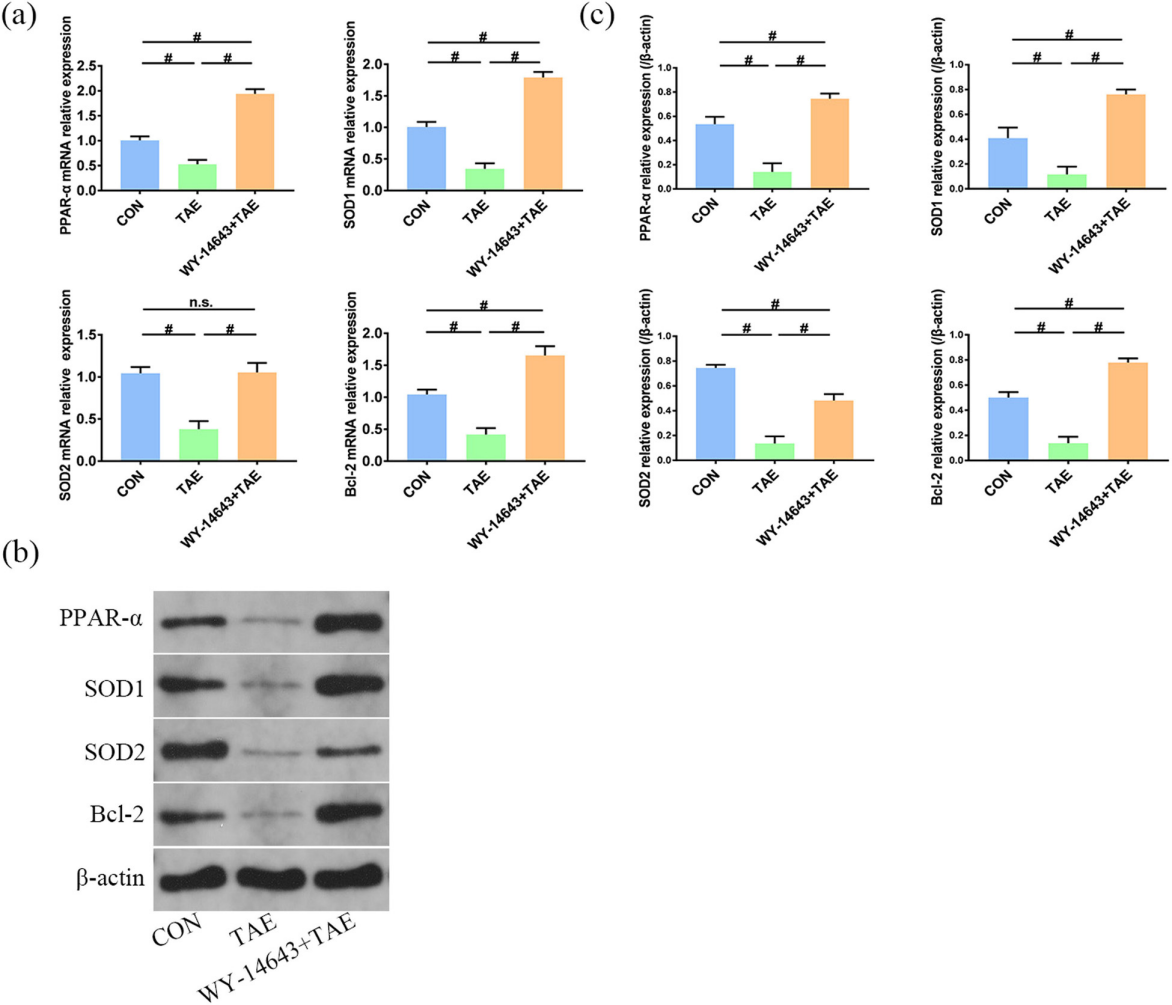
(a and c) Bar charts represent the relative gene and protein expression in non-tumorous liver tissues among the different experimental groups. (b) Representative Western blotting demonstrates protein expression among the different experimental groups: peroxisome proliferator-activated receptor-α, superoxide dismutase 1, superoxide dismutase 2, B-cell lymphoma-2 (*n* = 3). ^#^Significant (*P* < 0.05), Not significant (n.s., *P* > 0.05).

RT-qPCR was performed to observe the relative gene expressions of PPAR-α, SOD1, SOD2, and Bcl-2. Compared with the control group, the expression of these genes significantly decreased in the TAE group (*P* < 0.05) and significantly increased in the combined treatment group (*P* < 0.05). Furthermore, compared with the control group, the mRNA expression of PPAR-α, SOD1, and Bcl-2 was significantly increased in the combined treatment group (*P* < 0.05), while the SOD2 level changed slightly (*P* > 0.05, Figure 5a). These results reveal that WY-14643 can effectively activate PPAR-α, which may upregulate the expressions of SOD1, SOD2, and Bcl-2 by regulating gene transcription.

### 3.9 Western blotting to analyze PPAR-α, SOD1, SOD2, and Bcl-2 protein expression

Compared to the control group, the expressions of PPAR-α, SOD1, SOD2, and Bcl-2 significantly decreased in the TAE group (*P* < 0.05). However, compared with the TAE group, the protein expression was significantly increased in the combined treatment group (*P* < 0.05). Furthermore, compared to the control group, the expressions of PPAR-α, SOD1, and Bcl-2 significantly increased in the combined treatment group, while the expression of SOD2 significantly decreased (*P* < 0.05, Figure 5b and c). These results further demonstrate the antioxidant and anti-apoptotic capacity of PPAR-α after TAE.

## 4 Discussion

PLC is the fourth most common malignant cancer and the second-leading cause of cancer-related deaths in China [21,22]. Owing to delayed diagnosis, TAE has become the main treatment option for the majority of PLC patients [23]. However, embolic agents inevitably block blood vessels that supply non-tumorous tissues, which can lead to ischemia-reperfusion injury. Consequently, this can cause oxidative stress by producing a large amount of ROS and inflammation, ultimately resulting in liver damage [24,25,26]. Following TAE, we found that liver function markers, liver histopathology score, and apoptosis index significantly increased, suggesting that the adjacent liver tissue was damaged.

Elevated ROS mediates lipid peroxidation, leading to the production of MDA. Therefore, increased MDA levels reflect an increase in ROS and oxidative stress damage. Excessive ROS can impair mitochondrial DNA and ultimately cause irreversible damage to the organelles. SOD, GSH-PX, and CAT are natural free radical scavenging systems that maintain cellular equilibrium [27,28,29]. TNF-α promotes inflammation, induces the release of inflammatory cytokines, and promotes cell necrosis [30]. NF-κB is a multiprotein complex of transcriptional factors that induces the production of pro-inflammatory mediators leading to inflammation [31]. Our results showed that the expression of MDA, TNF-α, and NF-κB significantly increased after TAE, while SOD, GSH-Px, and CAT activities significantly decreased. Taken together, these results suggest the development of oxidative stress and inflammation in non-cancerous liver tissues.

In the combined treatment group, the apoptosis index was significantly decreased, the number of necrotic and apoptotic cells was significantly reduced, both ALT and AST levels significantly decreased, and liver damage was improved. In agreement with previous reports, our results imply that pretreatment with PPAR-α agonist WY-14643 can alleviate liver tissue injury [32]. PPAR-α was previously demonstrated to exhibit hypolipidemic effects as well as anti-inflammatory, anti-fibrotic, and anti-oxidative stress functions [33]. In the inactive state, PPAR complexes with RXR to form a heterodimer that binds to co-repressor proteins. Upon the binding of PPARs to ligands, the co-repressor proteins are released, and co-activator proteins are recruited. The activated PPAR/RXR-co-activator complex subsequently binds to specific DNA sequences or PPAR response elements (PPRE), leading to the downstream transcriptional activation of target genes [34]. SOD is divided into SOD1, SOD2, and SOD3, of which the former two are PPAR-α-regulated genes [35]. It was previously proposed that yeast becomes more susceptible to oxidative stress upon SOD1 and SOD2 deficiency, especially SOD1 [36,37]. Additionally, the Bcl protein family mediates the intrinsic mode of apoptosis. Thus, Bcl-2 is an important anti-apoptotic protein [38]. MDA content was significantly reduced in the combined treatment group, while the SOD, GSH-Px, and CAT activities were significantly increased. Furthermore, the expression of SOD1, SOD2, and Bcl-2 significantly increased upon WY-14643 pretreatment at the protein and mRNA levels. The abovementioned results suggest that the PPAR-α agonist, WY-14643, can enhance the antioxidant capacity, reduce apoptosis as well as necrosis, and improve liver function by enhancing the activity of antioxidant enzymes and upregulate the expressions of SOD1, SOD2, and Bcl-2 to inhibit oxidative stress and apoptosis. Furthermore, ROS inhibits the activity of the phosphatidylinositol 3-kinase (PI3K) signaling pathway, decreases the level of Bcl-2, promotes the release of cytochrome C from mitochondria, and consequently activates caspase-3, leading to cell apoptosis [39]. It is plausible to speculate that activated PPAR-α can enhance the antioxidant capacity and reduce ROS to upregulate the expression of Bcl-2, thereby suppressing apoptosis. Interestingly, the anti-inflammatory properties of palmitoylethanolamide are attributed to its ability to directly antagonize the NF-κB pathway via the selective activation of PPAR-α receptors [40]. Our results demonstrated that the expressions of NF-κB and TNF-α were significantly decreased in non-cancerous liver tissue of the combined treatment group. This implies the inhibitory effect of PPAR-α on the NF-κB pathway in the rabbit liver cancer model following TAE. Through tyrosine phosphorylation, hydrogen peroxide (H_2_O_2_) affects the degradation of the nuclear factor-enhancing kappa light chains of activated B cells (IκB-α), an NF-κB inhibitor [41]. Following WY-14643 pretreatment, the activity and expression of antioxidant enzymes in the non-cancerous liver tissue were significantly increased. Therefore, we speculate that PPAR-α could inhibit the NF-κB pathway by enhancing the antioxidant capacity and reducing ROS production. As a result, the inhibition of NF-κB signaling pathway reduces the secretion of proinflammatory cytokines, including TNF-α, which attenuates the role of its receptors (TNFR1) [42]. Recent studies have reported that PPAR-α is involved in the malignant progression of tumors. Fenofibrate, a PPARα agonist, can inhibit the growth of gliomas [43], while NF-κB modulates tumor formation and progression by inducing the expression of oncogenes involved in proliferation, survival, angiogenesis, and metastasis [44]. Silva-Gomez et al. [45] revealed that pirfenidone, a ligand/agonist of PPARγ, can prevent HCC development via modifying NF-κB p65/p50 signaling and translocation, preventing inflammation, and increasing p53 activity as well as caspase 3 activation. Compared to the TAE group, we observed that the expression of NF-κB was significantly decreased in the cancerous tissues of the combined treatment group, thus we speculate that PPAR-α agonist WY-14643 has an inhibitory effect on liver cancer following TAE. Taken together, PPAR-α appears to be a promising therapeutic target for liver diseases. Nevertheless, future studies will be dedicated to dissect the underlying molecular mechanism.

WY-14643 is an effective PPAR-α agonist that is often used in experimental research [46]. For instance, Li et al. demonstrated that WY-14643 mainly activates PPAR-α of mice hepatocytes [47]. Our experimental model showed that the expression of PPAR-α in the TAE group was significantly reduced but significantly increased following WY-14643 pretreatment. Taken together, we believe that WY-14643 can effectively activate PPAR-α in rabbit normal liver tissues. However, it is noteworthy that an excessively large dosage of WY-14643 has been reported to cause liver and kidney toxicity [48]. Therefore, adjusting the dosage of WY-14643 is critical to obtain a favorable outcome.

## 5 Conclusion

In conclusion, our research findings provide a theoretical basis for the prevention, treatment, and prognosis of liver diseases in clinical settings. The results confirmed that WY14643 could activate PPAR-α, which subsequently enhances the antioxidant, anti-inflammatory, and anti-apoptotic pathways in non-cancerous liver tissue and may be critical for tumor suppression. Overall, this work demonstrated that PPAR-α significantly alleviated liver injury in the established rabbit liver cancer model.
